# Physicochemical Characterization and Antioxidant Properties of Cellulose-Rich Extracts Obtained from Carob (*Ceratonia siliqua* L.) Pulp for Preparation of Cellulose-Rich Gels

**DOI:** 10.3390/gels11020145

**Published:** 2025-02-18

**Authors:** Bernat Llompart, Esperanza Dalmau, Mónica Umaña, Antoni Femenia

**Affiliations:** Department of Chemistry, University of the Balearic Islands, Ctra. Valldemossa, Km 7.5, 07122 Palma, Spain; bernat.llompart3@estudiant.uib.cat (B.L.); esperanza.dalmau@uib.es (E.D.); monica.umana@uib.es (M.U.)

**Keywords:** carob, *Ceratonia siliqua*, cellulose-rich gels, antioxidants, by-products

## Abstract

The carob tree (*Ceratonia siliqua* L.) is a defining species of the Mediterranean region, and its fruit, the carob pod, has seen a notable increase in economic interest in recent years, primarily due to the production of locust bean gum (E410), a widely used food additive derived from the seeds. The remainder of the fruit, the carob pulp, comprises 80–90% of the fruit’s weight and is typically considered a by-product, with its primary application being in animal feed. This study focused on obtaining cellulose-rich extracts from selected carob varieties cultivated in the Mediterranean region. A comprehensive physicochemical characterization of these cellulose-rich fractions was conducted, including the assessment of their antioxidant properties, specifically total phenolics and antioxidant capacity measured by the FRAP, ABTS, and CUPRAC methods. The findings reveal that carob pulp is an excellent source of carbohydrates, including soluble sugars, which constitute 33–45% of the pulp’s fresh weight, depending on the variety, and cell wall polysaccharides. The cell wall polymers, with cellulose as the predominant component, account for approximately 45% of the fresh pulp weight. Notable amounts of other polysaccharides, such as pectins and hemicelluloses, were also identified. Among the studied varieties, *Bugadera* and *Rotjal* stood out as exceptional sources of cellulose-rich extracts. Carob pulp was also found to be rich in antioxidant compounds, reflected in its high antioxidant capacity. In particular, the *Bugadera* variety, grown under irrigated conditions, exhibited a significant concentration of phenolic compounds (24.4 mg gallic acid equivalents per gram of pulp) and high antioxidant activity across all methods used, with ABTS measurements reaching up to 391.5 mg Trolox equivalents per gram of pulp. In conclusion, these results underscore the significant potential of carob pulp as a source of valuable cellulose-rich extracts, offering applications beyond its traditional use as animal feed. By exploring these new possibilities, the economic and environmental sustainability of carob cultivation could be greatly enhanced, contributing to the broader valorization of this iconic Mediterranean fruit.

## 1. Introduction

The carob tree (*Ceratonia siliqua*), a member of the legume family, is a perennial plant native to the Mediterranean region. It is highly valued for its fruit, the carob pod, which has multiple uses in the food industry and offers significant health benefits [1]. The cultivation of carob trees plays a particularly vital role in Spain, which is one of the world’s largest producers of carob, with the Balearic Islands playing a leading role in its cultivation. In this region, the carob tree is not only a source of economic revenue but also a key element in preserving traditional agricultural landscapes [2].

Carob seeds, or “carob beans”, are the most valuable part of the fruit and are extensively used in the food industry to produce locust bean gum (LBG, E410), a thickening and gelling agent derived from the seed endosperm. In contrast, the pulp of the carob, which constitutes the majority of the fruit, is often discarded as a low-value byproduct despite its potential as a source of valuable nutrients and functional ingredients [3].

Carob pulp is rich in natural sugars (40–50%), dietary fiber, and bioactive compounds, such as polyphenols and antioxidants. It contains negligible fat, making it an appealing ingredient for health-conscious consumers [3,4,5]. Despite its high content of dietary fiber, there has been no comprehensive characterization of the polysaccharides present in carob pulp. Understanding the structure and properties of these molecules would open new avenues for their application in the food, pharmaceutical, and other industries. Such studies could enhance the value of carob as a versatile raw material and support its broader use in innovative products. The most abundant structural polysaccharides in vegetable cell walls are cellulose, hemicellulose, and pectins.

Hemicelluloses, in contrast, are polysaccharides with backbones made up of glucose, mannose, and/or xylose connected by β-(1 → 4) linkages. They associate with cellulose, proteins, and lignin, mainly through non-covalent interactions, such as hydrogen bonds [6,7].

Pectins are heteropolysaccharides primarily composed of homogalacturonans, with a backbone of D-galacturonic acid residues. Rhamnogalacturonan I features a galacturonic acid backbone interspersed with L-rhamnose residues linked to side chains of arabinose and galactose. Pectin extracts may also include xylogalacturonan and the less common rhamnogalacturonan II, a highly branched structure [8].

Polysaccharides, such as pectin, cellulose, and hemicellulose, play an important role in various food industry applications. These polysaccharides are valuable for their ability to form gels and act as stabilizing agents in food products [9]. Edible gels made from polysaccharides are widely used to improve texture, create low-calorie desserts, and stabilize emulsions in dairy and non-dairy products [10]. The use of carob-derived polysaccharides as natural thickeners and gelling agents aligns with the growing demand for clean-label ingredients, which are increasingly preferred by consumers seeking minimally processed and sustainable products [11].

On the other hand, carob pulp is rich in bioactive compounds, particularly polyphenols, which contribute to its antioxidant properties. The predominant phenolic compounds identified in carob pulp include gallic acid, catechin, and various flavonoids. These compounds are known for their ability to neutralize free radicals, thereby reducing oxidative stress and potentially lowering the risk of chronic diseases [12].

Several studies have investigated the use of carob pulp in the food industry, showcasing its functional and nutritional benefits. Carob flour has been successfully incorporated into bread formulations, enhancing dough characteristics, texture, and moisture retention [13]. Its high sugar content, mainly sucrose, makes it suitable for the production of carob syrup, a natural sweetener [14], while its similarity in appearance and color to chocolate has led to its use as a caffeine-free substitute [15]. Additionally, aqueous extracts of carob pulp, rich in bioactive compounds like tannins, flavonol glycosides, and gallic acid, have been utilized in developing functional foods due to their antioxidant properties [16].

The chemical composition of carob pods, including sugar, fiber, and polyphenol content, can vary significantly depending on the cultivar, region of cultivation, and agricultural practices [12,17]. This variability directly impacts the quality and applications of carob in the food industry.

The objective of this study is to characterize the pulp of carob (*Ceratonia siliqua* L.) varieties native to the Balearic Islands, with a specific focus on the polysaccharide-rich fraction. By conducting a comprehensive physicochemical analysis and evaluating its antioxidant properties, this research aims to explore the potential of these extracts for the development and preparation of cellulose-rich gels. The findings aim to highlight innovative applications of Balearic carob varieties in the food and health industries.

## 2. Results and Discussion

### 2.1. Physicochemical Characterization of the Pulp from Different Carob Varieties

Table 1 shows the percentages corresponding to each of the main parts of the carob fruit, namely the seed as the primary product and the pulp as a by-product, for each of the analyzed carob varieties [18].

The selected carob varieties showed pulp or by-product percentages ranging from 82.5% for the *Valenciana* variety to 89% for the Franco variety (Table 1). Overall, these values are similar to those reported by Adam [19], who observed average pulp contents of 90% by weight relative to the total fruit.

These high pulp values underscore the importance of studying and evaluating potential applications for this by-product, such as the production of cellulose-rich gels, particularly when the fruit is harvested exclusively for its seeds. To this end, the study first focused on a general characterization, followed by a detailed analysis of dietary fiber and antioxidant compounds. Additionally, the research was conducted on six previously mentioned varieties to assess the potential influence of varietal differences on the analyzed parameters.

Table 2 shows the results of moisture content, pH, acidity, soluble sugars, and cellulose-rich extract (CRE) content of different carob pulp varieties [18].

The average moisture content of the carob pulp was approximately 12.8 ± 0.9% (g H_2_O/100 g pulp), a value that is higher than the moisture content previously reported by Brassesco et al. [1], who found a moisture content of approximately 6%. Significant differences (*p* < 0.05) were found between the *Valenciana* and *Fulla de raó* varieties, with the latter having 3.2% less moisture content.

The pulp of the different carob varieties was characterized by a slightly acidic pH, with an average value of 5.1 ± 0.1, which is similar to the pH value determined by Tounsi et al. [20]. Except for the *Valenciana* and *De la mel* varieties, the other varieties showed significant differences (*p* < 0.05) in pulp pH, with the *Valenciana* variety having the highest pH value and the *Bugadera* variety having the lowest pH. The carob pulp samples showed an average acidity value of 3.3 ± 0.2 g citric acid/100 g pulp, a value similar to that determined by Benchikh et al. [21], who reported an acidity of 2.7 ± 0.1 g citric acid/100 g pulp in carob samples from different varieties originating in Algeria. Significant differences in acidity were found among the varieties, with *De la mel* having the highest acidity content and *Rotjal* the lowest. As shown in Table 2, no direct relationship was found between pH and acidity, as might be expected. The relationship between pH and acidity (expressed as citric acid content) in carob pulp is not always straightforward, as pH is a logarithmic measure of hydrogen ion concentration, whereas acidity quantifies the total amount of acid present in the sample. Although higher acid content would suggest lower pH values, the pH of the pulp can be influenced by several factors beyond organic acid concentration. These factors include the presence of buffering compounds, such as salts and other basic components, which can mitigate the acidifying effect of the acid. Additionally, the composition of different carob varieties may lead to various interactions between these compounds, potentially altering the pH without significantly affecting the total acidity. Furthermore, the ionization of acids and the presence of other organic and inorganic substances in the pulp could further complicate this relationship. Therefore, while pH and acidity are related, they should not be expected to change in a directly proportional manner [22].

The soluble sugar content was relatively high for all the samples analyzed. The average value for soluble sugars was approximately 38.5 ± 1.2% (expressed as g sucrose/100 g pulp). All obtained values fall within the range reported by Khalifa et al. [23], which was from 32.6 to 45.4 g sucrose/100 g pulp, in a study of different carob varieties from northern Morocco.

The extraction of CRE for each sample allows the determination of the chemical composition of the various polysaccharides forming the cell walls of carob pulp, as well as the functional properties related to these compounds. As shown in Table 2, the yields of the samples ranged from 40.9 g CRE/100 g pulp for the *Fulla de raó* variety to 49.1 g CRE/100 g pulp for the Rotjal variety. The average CRE yield was approximately 44.5 ± 2.1 g CRE/100 g pulp, a slightly higher value than that reported by Nasar-Abbas et al. [24], who found a value of 40 g CRE/100 g pulp. It is worth noting that significant differences were observed between the varieties analyzed in this study (*p* < 0.05), with the Bugadera variety showing the lowest CRE content, while no significant differences were found among the other varieties.

### 2.2. Characterization of the Carob Cellulose-Rich Fraction (CRF)

#### 2.2.1. Sugar Content

The cellulose-rich extract (CRF) contains all the polysaccharides present in the carob pulp samples, particularly the polymers that form the cell wall, as well as lignin [25]. Polysaccharides are composed of sugar units, known as monomers, which are linked through glycosidic bonds. Through hydrolysis, the individual content of each sugar comprising the various polysaccharides was determined. The results obtained, expressed as mg sugar/g CRF, are shown in Table 3 [18].

**Figure 1:**
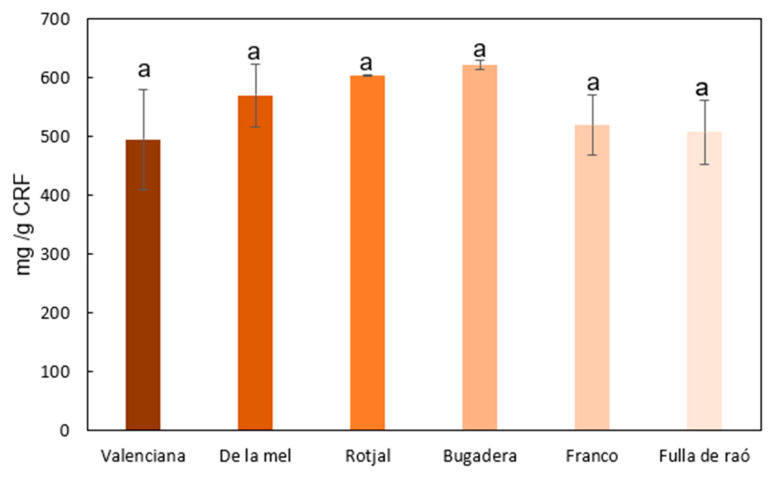
Total polysaccharide content in the CRFs of different carob pulp varieties. Different letters indicate significant differences (*p* < 0.05).

In general, significant amounts of glucose were observed, suggesting the presence of cellulose. Additionally, a high presence of pectic polysaccharides or pectins can be deduced from the substantial amount of uronic acids, particularly galacturonic acid [25]. On the other hand, rhamnose, arabinose, and galactose, though detected in lower quantities compared to uronic acids, are also components of pectins. The remaining sugars detected, particularly xylose, and to a lesser extent mannose and fucose, indicate the presence of significant amounts of hemicelluloses [26]. Glucomannans and fucose-related polymers were not detected in significant amounts in the analyzed carob pulp samples. The results indicate that the main hemicelluloses present are primarily composed of xylans, which align with the compositional profile of the cellulose-rich fractions obtained. From the individual sugar quantities detected in the CRF, the total polysaccharide content present in the different samples was estimated. Figure 1 shows the total polysaccharide content (mg/g CRF) for each of the different samples analyzed. As can be seen, no significant differences (*p* > 0.05) were found in the polysaccharide content of the different CRFs. The average total polysaccharide content in the CRF samples was 552.7 ± 37.8 mg/g CRF. Varieties such as *Fulla de raó* and *Valenciana* fall below this average, while other varieties such as Rotjal and Bugadera present values above it.

The high polysaccharide content derived from carob pulp, with a value of approximately 25% of the fresh pulp weight, indicates a very high dietary fiber content. This value closely matches the one presented by Papaefstathiou et al. [27] for three carob varieties originating from Cyprus, with a dietary fiber content of 25.7%.

#### 2.2.2. Polysaccharide Characterization

**Figure 2:**
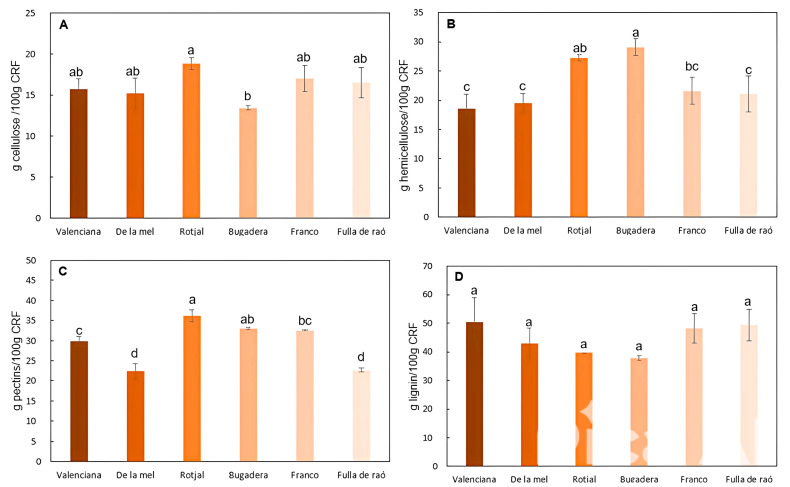
Cellulose (**A**), hemicellulose (**B**), pectins (**C**), and lignin (**D**) content (expressed in g/100 g CRF) in different carob varieties. Different letters indicate significant differences (*p* < 0.05).

Figure 2 illustrates the cellulose, hemicellulose, pectin, and lignin content in the cellulose-rich fractions (CRFs) of six different carob varieties.

Cellulose is the most abundant structural polymer in plants, composed of glucose units linked by β-1,4 glycosidic bonds [28,29]. As shown in Figure 2A, the cellulose content of the CRF from the studied carob varieties ranged from 13% to 19%. Statistically significant differences (*p* < 0.05) were observed among the varieties, with the *Rotjal* variety exhibiting the highest cellulose content and the *Bugadera* variety the lowest. The average cellulose content across all varieties was approximately 16 ± 1% of the CRF. This corresponds to a cellulose content of 5.6 to 9.3 g per 100 g of fresh carob pulp. These values are comparable to the cellulose content of fruits, such as carrots (10.1%), apples (8.8%), and tomatoes (8.6%), and are slightly lower than that of cucumbers (16.1%) [30].

Hemicelluloses are highly branched polysaccharides composed primarily of xylose, fucose, mannose, and glucose. These polymers mainly include xyloglucans and xylans. Figure 2B illustrates the total hemicellulose content in the CRFs obtained from the various carob varieties. The hemicellulose content ranged from 18.5% to 29.0% of the CRF. Among the studied varieties, *Bugadera* exhibited the highest hemicellulose content, while *Valenciana* and *De la Mel* had the lowest levels. On average, the hemicellulose content in the CRFs corresponded to 10.2 ± 0.8 g/100 g of fresh pulp. This value exceeds the hemicellulose content reported by Szymańska-Chargot et al. [30] for fruits, such as carrots, tomatoes, cucumbers, and apples, which averaged 5.2 g/100 g of fresh sample. The elevated hemicellulose content in the CRFs is likely attributable to the presence of xylans, which are deposited in the cell wall along with lignin.

Pectins are complex polysaccharides characterized by a high degree of branching. These polymers are primarily composed of galacturonic acid units, with smaller amounts of galactose, rhamnose, and arabinose [31]. Pectins are notable for their bioactivity, offering various health benefits, including antitumor properties [32]. Figure 2C illustrates the total pectin content in the CRFs of the carob varieties analyzed. Among the polysaccharides studied, pectins were the most abundant in the cell walls of the carob varieties. The total pectin content ranged from 22.5% to 36.0% of CRF, with an average of 28.5 ± 9.4% CRF. The *Rotjal* variety showed the highest pectin content, with 17.8 ± 0.8 g/100 g of fresh pulp, whereas the *De la Mel* and *Fulla de Raó* varieties displayed the lowest values, the latter being 10.1 ± 0.2 g/100 g of fresh pulp. The pectin content in carob pulp was lower than the range reported for banana peels (16–24%) by Khamsucharit et al. [33] and comparable to the pectin yield (19.2%) reported for orange peels by Prakash et al. [34]. Structural characteristics of pectins can be inferred through specific monomeric relationships (Equations (1)–(3)), with results shown in Table 4 [18]. The linearity, chain number, and chain length of pectins were analyzed [31]. The *Rotjal* and Franco varieties exhibited the highest linearity values, while the *De la Mel* and *Fulla de Raó* varieties showed the lowest. Regarding the number of chains, *Rotjal* and *Franco* had the highest values, whereas *De la Mel* displayed the lowest. No significant differences in chain length were observed among the varieties (*p* > 0.05). Additionally, the degree of methyl esterification (DME) of pectins was determined via FTIR-ATR, with values ranging from 45% to 70%. The *Rotjal* variety had the highest DME, while the *Franco* variety had the lowest. With an average DME of 58.3%, all varieties except *Franco* can be classified as high-methoxyl (HM) pectins. HM pectins are polysaccharides with a DME above 50%, indicating that more than half of their carboxyl groups are esterified with methanol [31]. They are uniquely suited to form gels in the presence of high sugar concentrations (typically above 55%) and low pH values (2.8–3.6).

Gelation involves hydrophobic interactions between pectin chains and sugar molecules, stabilized by hydrogen bonds. These properties make HM pectins indispensable in the food industry, particularly for producing jams, jellies, and confectionery products requiring high sugar content. They are also used for creating edible coatings due to their film-forming capabilities, contributing to texture and stability in sugary, acidic food products [35].

The results for lignin content in the different samples, expressed as a percentage of CRF, are shown in Figure 2D. The average lignin content across the CRF samples was 44.7 ± 3.8% of CRF. Varieties such as *Fulla de Raó* and *Valenciana* had lignin levels above this average, while others, including *Rotjal* and *Bugadera*, presented lower values. It is noteworthy that no significant differences were observed between the varieties (*p* > 0.05). The average lignin content per 100 g of fresh carob pulp was 19.9 ± 1.9 g lignin/100 g fresh pulp. This value is higher than the 14.0 ± 0.6 g lignin/100 g fresh sample reported for banana peels by Velásquez-Arredondo et al. [35].

#### 2.2.3. Functional Properties

**Figure 3:**
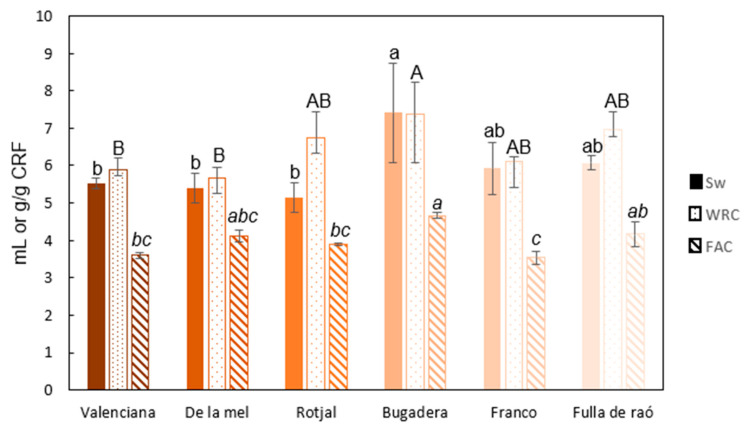
Functional properties determined for the CRF of different carob varieties. Sw: swelling; WRC: water retention capacity; FAC: lipid adsorption capacity. Different letters indicate significant differences *p* < 0.05.

The chemical composition and structural characteristics of polysaccharides can greatly influence functional properties related to hydration processes, such as swelling (Sw) and water retention capacity (WRC), or the adsorption of organic molecules, such as fat adsorption capacity (FAC) [36]. Figure 3 shows the swelling capacity, water retention capacity, and fat adsorption capacity of the cellulose-rich fractions (CRFs) from different carob varieties.

The swelling values of the analyzed carob varieties ranged from 5 to 7.5 mL/g CRF, with significant differences observed among the varieties (*p* < 0.05). The *Bugadera* variety exhibited the highest swelling capacity, while the *Rotjal* variety showed the lowest. The average swelling capacity (Sw) was 5.9 mL/g CRF, a value lower than the 11.4 mL/g CRF reported by Petkova et al. [37] for various Bulgarian carob. Swelling capacity is a critical parameter for evaluating the potential of a cellulose-rich extract to form gels. Previous studies have suggested that optimal swelling values for forming well-structured three-dimensional gels typically range between 10 and 20 mL/g of dry extract. These values reflect a high interaction of the material with water, which is essential for food or biomedical applications requiring flexible and highly hydrated gels [38]. However, the swelling values obtained in this study, ranging between 5 and 7.5 mL/g, while lower, are suitable for the formation of more compact and rigid gels. This lower swelling capacity may be associated with the presence of components such as hemicelluloses or lignin, which limit water absorption capacity but contribute to the structural stability of the gel network. Gels formed with these extracts would be less prone to collapse or water loss, which is advantageous in specific applications such as the fabrication of edible coatings or the production of dense materials for 3D printing, where high structural definition is required. Therefore, although the values obtained are below those considered ideal for highly hydrated gels, they are suitable for developing gelled systems with improved mechanical properties and stability under adverse environmental conditions [39].

The water retention capacity (WRC) is determined by the amount of water retained inside a sample after being subjected to an external force, typically through a centrifugation process. As shown in Figure 3, the values ranged between 5.5 and 7.5 g H_2_O/g dry matter. The *Bugadera* variety exhibited the highest WRC, while the *De la Mel* variety had the lowest capacity. The water retention capacity of carob flour in a study by Petkova et al. [37], with different carob varieties from Bulgaria, was 1.4 ± 0.2 g H_2_O/g dry matter, which is lower than the value obtained in this study, where the average value was approximately 6.5 g H_2_O/g dry matter. Rodríguez-González et al. [36] highlighted that values between 7 and 12 g/g are common for extracts derived from plant residues with good gelling capacity.

Finally, the lipid adsorption capacity (FAC), defined as the amount of oil that the sample can retain after being subjected to a centrifugation process, was determined. FAC has been directly linked to the inhibition of toxicological and carcinogenic factors, as well as the reduction in blood cholesterol levels [40]. The values obtained ranged from 3.5 to 4.5 g oil/g CRF (Figure 3), with the *Bugadera* variety showing the highest lipid adsorption capacity, while the *Franco* and *Valenciana* varieties exhibited lower values. The differences in FAC values among the carob samples were statistically significant (*p* < 0.05). The average FAC value was approximately 4.0 g oil/g CRF, a value similar to that reported by Petkova et al. [37]. For gel formation from cellulose-rich extracts, lipid retention capacity (FAC) is an important property, especially in applications where gels must interact with or encapsulate lipophilic compounds such as oils, fats, or bioactive compounds. Although the optimal FAC values may vary depending on the type of extract and the specific application, values ranging from 2 to 6 g of lipids per g of extract are generally considered optimal for gel formation applications [41].

#### 2.2.4. Antioxidants Compounds

Another interesting aspect for the potential valorization of carob byproducts is understanding their antioxidant properties.

The results obtained for the total phenolic content (TPC) and the antioxidant capacity of the different carob varieties, evaluated using the ABTS, FRAP, and CUPRAC methods, are presented below (Table 5).

According to the literature, carob pulp is rich in polyphenolic compounds, which can exert a beneficial effect on human health [42]. The total phenolic content (TPC) in the carob pulp extracts showed significant variability among the different analyzed varieties, with values ranging from 30 mg gallic acid/g CRF (*Rotjal*) to 68 mg gallic acid/g CRF (*Bugadera*). The *Bugadera* variety exhibited the highest phenolic content, while *Rotjal* showed the lowest value. Other varieties, such as *De la mel* and *Fulla de raó*, had intermediate levels, with values close to 38 mg gallic acid/g CRF. These results were lower than those found by Petkova et al. [37], who reported phenolic content in carob polysaccharide extracts of 115 ± 16 mg gallic acid/g CRF for different varieties from Bulgaria. These data suggest that *Bugadera* has an antioxidant potential closer to the varieties studied by Petkova et al. [37], reinforcing the potential of this variety for antioxidant applications.

Regarding the results obtained using the FRAP method (Table 5), it is noteworthy that *Bugadera* and *De la mel* presented the highest antioxidant capacities, with values of approximately 50 mg Trolox/g CRF. On the other hand, the *Rotjal* variety exhibited the lowest antioxidant capacity with a value of 21 mg Trolox/g CRF. The average value of this study (35 ± 10 mg Trolox/g CRF) was similar to that reported by Saci et al. [43] for two Algerian carob varieties, which showed an antioxidant capacity, determined by the FRAP method, of 32 ± 17 mg Trolox/g CRF.

The antioxidant capacity, measured by the ABTS method, ranged widely from 480 to 1070 mg Trolox/g CRF, with *Bugadera* exhibiting the highest antioxidant capacity. On the other hand, *Franco* and *Rotjal* varieties showed the lowest antioxidant capacities. Significant differences (*p* < 0.05) were observed between *Bugadera* and the other varieties. The average value of this study (700 ± 200 mg Trolox/g CRF) was higher than that reported by Rtibi et al. [44] for various carob varieties from Tunisia, which showed an ABTS-measured antioxidant capacity of 580 ± 10 mg Trolox/g CRF.

Finally, in the determination of antioxidant capacity using the CUPRAC method, it was again observed that *Bugadera* and *De la mel* exhibited the highest antioxidant capacities, while *Rotjal* presented the lowest capacity. In this case, a wide range of antioxidant capacities was observed for the CRF of the different carob varieties analyzed, ranging approximately from 250 to 700 mg Trolox/g CRF. The results presented in this study were also significantly higher than those reported by Saci et al. [43], with an antioxidant capacity, determined by the CUPRAC method, of 188 ± 5 mg Trolox/g CRF.

In summary, the results obtained for total phenolic content and antioxidant capacity of the different carob varieties analyzed show clear variability, with *Bugadera* standing out due to its high values in all measurements. These results are consistent with previous studies and emphasize the relevance of carob extracts, especially *Bugadera*, for applications where antioxidant capacity is key. While the *Bugadera* variety exhibited the highest total phenolic content (TPC), the antioxidant activities measured by the ABTS, FRAP, and CUPRAC methods showed significant variability across the analyzed varieties. This finding suggests that the antioxidant capacity is not solely dependent on the quantity of phenolic compounds but also on their specific composition and structure. For instance, certain phenolic compounds may exhibit higher reactivity in specific assays, while others may have limited activity. Additionally, synergistic or antagonistic interactions between phenolic compounds and other bioactive components, such as polysaccharides or proteins, could influence the measured antioxidant capacity. These results highlight the importance of characterizing the specific phenolic profile and understanding its functional contributions rather than relying solely on total phenolic content as an indicator of antioxidant potential.

It is important to acknowledge that the properties analyzed in this study correspond exclusively to carob pods harvested in 2022. This may limit the generalizability of the findings, as environmental factors such as climate conditions, agricultural practices, and soil characteristics can vary annually and potentially influence the physicochemical and antioxidant properties of carob pulp. Future studies should consider including samples from multiple harvest seasons to evaluate the impact of annual variations on the properties of cellulose-rich extracts.

## 3. Conclusions

This study highlights the significant potential of carob (*Ceratonia siliqua* L.) pulp as a source of functional compounds, particularly cellulose-rich extracts, with applications in various industries. The analysis of six carob varieties native to the Balearic Islands revealed important varietal differences in physicochemical and structural properties, demonstrating the influence of genetic factors on pulp composition.

Key findings include the high content of polysaccharides in the cellulose-rich fraction (CRF), with notable proportions of cellulose, hemicelluloses, and pectins. The Rotjal variety exhibited the highest cellulose content, indicating its suitability for applications requiring robust gel structures, while Bugadera showed a higher hemicellulose content, contributing to specific functional properties. Additionally, the antioxidant potential of the Bugadera variety stood out, making it particularly valuable for applications where antioxidant capacity is critical.

Pectins in the carob pulp demonstrated significant structural variability among the varieties, with high degrees of methyl esterification, especially in Rotjal and Franco, supporting their potential in food gel formulation and edible coatings. These findings underscore the relevance of varietal selection to optimize compound extraction and tailor the functional properties of carob pulp for specific applications.

In conclusion, the results confirm the versatility of carob pulp as a valuable by-product, with significant potential for the preparation of cellulose-rich gels and other innovative applications. Further studies incorporating samples from multiple harvest seasons are recommended to assess the influence of annual variations on the properties of these extracts and to expand their applicability in industrial processes.

## 4. Materials and Methods

### 4.1. Physicochemical Characterisation of the Pulp from Different Carob Varieties

Six different carob varieties were analyzed. All these samples were collected in 2022 at the Sa Mata estate located in Randa (municipality of Algaida, Spain) and the experimental field Son Mulet (Llucmajor, Spain). Table 6 shows a description of the six different carob varieties studied in this work.

The percentage (by weight) of pulp and seed in each carob variety was obtained by weighting the pulp and seed separately.

The carob pulp was ground using an IKA M20 laboratory mill (Barcelona, Spain) and then sieved to obtain flour with a particle size smaller than 0.25 mm. This flour was used to carry out the chemical characterization.

#### Moisture, pH, Acidity, Soluble Sugars and Cellulose-Rich Fraction

The moisture content of the different carob varieties was determined using a Denver Instrument IR 60 moisture analyzer (Madrid, Spain).

The pH was determined following the procedure described by [45]. In a beaker, 0.5 g of carob pulp or flour was weighed, and 2 mL of distilled water was added. The pH was then measured using a calibrated pH meter while keeping the mixture under constant agitation.

After obtaining the initial pH value, to calculate acidity, a buret containing 0.1 N sodium hydroxide was prepared, and the solution was added to the sample until a final pH of 8.1 was reached. Results are expressed as grams of citric acid per 100 g of the sample.

Soluble sugars were determined following the AOAC 932-14C procedure [46]. Approximately 1 g of carob flour was weighed, and 12 mL of distilled water was added. The mixture was dissolved at 40 °C for 15 min and then vacuum-filtered. Using a refractometer (Abbe 325 Zuzi, Barcelona, Spain), calibrated with distilled water, the reading was taken at 20 °C. Results were expressed as a percentage of sucrose (g sucrose/ 100 g of sample).

The cellulose-rich extract (CRE) was derived from carob flour by isolating the alcohol-insoluble residue (AIR) from various carob varieties. The method proposed by Femenia et al. [25] was followed. Approximately 20 g of each carob pulp was mixed with ethanol (1 L) to achieve a final concentration of 85%, and the mixture was homogenized (1 min at 13,000 rpm) using an Ultra Turrax (Heidolph DIAX 600, Schwabach, Germany). The mixture was boiled for 5 min to inactivate enzymes that could degrade the different polysaccharides of the cell wall. After 5 min, the sample was again homogenized (2 min, at 13,000 rpm) and boiled again for 1 min. Thereafter it was filtered using a cellulose-free glass fiber filter (Whatman GF-C, Scharlab SL, Barcelona, Spain). This was followed by two additional ethanol washes (approximately 100 mL each), with a final rinse using absolute ethanol. The sample was then filtered again and sequentially washed with absolute ethanol and acetone (about 100 mL each). The AIR yield was determined as the amount of CRF (g) per 100 g of carob pulp flour. The final product was obtained in powdered form.

### 4.2. CRE Characterization

#### 4.2.1. Sugar Content

The sugars, the basic units of the polysaccharides from the cell walls of the carob pulp, were released through a hydrolysis process (Saeman hydrolysis method) and subsequently converted into alditol acetates. The resulting monosaccharides (rhamnose (Rha), fucose (Fuc), arabinose (Ara), xylose (Xyl), mannose (Man), galactose (Gal), and glucose (Glc)) were separated and analyzed through gas–liquid chromatography, following the protocol outlined by Dalmau et al. [47]. Uronic acids were quantified colorimetrically as total uronic acid after hydrolyzing the samples for 1 h at 100 °C [48]. Briefly, for the hydrolysis of AIR samples, 1.2 mL/g of sulfuric acid/tetraborate was added, and the tubes were immediately cooled in crushed ice. The mixture was then vortexed to ensure proper agitation before being heated at 100 °C for 5 min. After heating, the samples were rapidly cooled in a water-ice bath, and 20 µL of m-hydroxydiphenyl reagent was added. The tubes were then shaken, and absorbance was measured at 520 nm using a Cary Bio 300 spectrophotometer (Varian, Palo Alto, CA, USA) within 5 min. Galacturonic acid, dissolved in saturated benzoic acid, served as the standard within a concentration range of 0–80 µg/mL.

#### 4.2.2. Polysaccharides and Lignin

Based on the composition of the polysaccharides in the cell wall, the contents of cellulose, pectin, and hemicellulose in the CRE were calculated from the mass (grams) of the monosaccharides, which were previously determined, using Equations (1)–(3) [49].

(1) $$\%\mathrm{Cellulose}=\frac{\;\mathrm{glc}\times 0.9}{\mathrm{CRE}}\times 100$$

(2) $$\%\mathrm{Pectin}=\frac{\mathrm{Rha}+\mathrm{Ara}+\mathrm{Gal}+\mathrm{UA}\;\;}{\mathrm{CRE}}\times 100$$

(3) $$\%\mathrm{Hemicellulose}=\frac{\mathrm{Fuc}+\mathrm{Xyl}+\mathrm{Man}+\mathrm{Glc}\times 0.1\;\;}{\mathrm{CRE}}\times 100$$

Finally, lignin was estimated by the difference between the material contained in the AIR and the sum of the total polysaccharides in the sample.

In the case of the pectins, parameters such as linearity, chain branching, and chain length were calculated from the molar ratios of sugars. The molar content of UA was divided by the remaining sugars that make up pectins (Rha, Gal, and Ara), which allows for determining the linearity of the pectins (Equation (4)). Rhamnogalacturonan (RG) chains are the most common in pectins, and since they are linked to the main chain by rhamnose units, the ratio of UA to Rha molar content will be inversely proportional to the number of chains (Equation (5)). The chain length was calculated by dividing the molar content of Gal and Ara by the moles of Rha (Equation (6)) [50,51].

(4) $$\mathrm{Lineality}=\frac{\;[\mathrm{UA}]}{\left[\mathrm{Rha}\right]+\left[\mathrm{Gal}\right]+[\mathrm{Ara}]}$$

(5) $$\mathrm{Number}\;\mathrm{of}\;\mathrm{side}\;\mathrm{chains}=\frac{\;[\mathrm{UA}]}{\left[\mathrm{Rha}\right]}$$

(6) $$\mathrm{Side}\;\mathrm{chains}\;\mathrm{lenght}=\frac{\left[\mathrm{Gal}\right]+[\mathrm{Ara}]\;\;}{\left[\mathrm{Rha}\right]}$$

The degree of methyl esterification (DME) of the pectins was determined by FTIR-ATR spectroscopy (Tensor 27 Bruker model, with a resolution of 4 cm^−1^, Billerica, MA, USA) following the methodology described by Manrique et al. [52]. Approximately 2 mg of the sample were placed in the instrument. The DME was quantified using the following equation.

(7) DM=124.7R+2.2013

where R was calculated as the ratio of the area of the peak at 1740 cm^−1^ over the sum of the area of the peak at 1740 cm^−1^ and at 1630 cm^−1^. Being 1740 cm^−1^ and 1630 cm^−1^ the absorbance bands for methyl-esterified and non-methyl-esterified carboxyl groups, respectively.

#### 4.2.3. Functional Properties

In this study, hydration-related properties were assessed, including swelling capacity (Sw), water retention capacity (WRC), and fat adsorption capacity (FAC).

To determine Sw, about 1 g of CRE (m_1_) was added to a test tube containing 10 mL of 1 M sodium phosphate buffer solution (pH 6.2) to replicate the pH conditions of different food systems. The initial sample volume (V_1_) was noted, and the mixture was left to rest for 24 h to achieve equilibrium, representing its highest swelling potential (V₂). After this duration, the volume of the swollen sample in the test tube was recorded [53]. The swelling capacity (Sw) was then calculated using Equation (8).

(8) $$\mathrm{Sw}=\frac{V_{2}-V_{1}}{m_{1}}$$

The WRC was determined by suspending approximately 1 g of CRE (m₂) in an excess of 1 M sodium phosphate buffer solution (pH 6.2) for 24 h. Afterward, the suspension was centrifuged at 1750× *g* for 25 min using an ALC 4218 centrifuge (Thermo Scientific, Milan, Italy). The solid and liquid phases were separated by decantation, and the solid phase was weighed (m₃). WRC was calculated using Equation (9).

Similarly, FAC was evaluated by putting a pre-weighed sample of CRE (m₄) in sunflower oil for 24 h. Following this, solid and liquid were separated through centrifugation (1750× *g* for 25 min) and decantation. The solid phase was then weighed (m₅), and FAC was determined using Equation (10) [54]

(9) $$\mathrm{WRC}=\frac{m_{3}-m_{2}}{m_{2}}$$

(10) $$\mathrm{FAC}=\frac{m_{5}-m_{4}}{m_{4}}$$

### 4.3. Antioxidant Compounds

Total phenolic compounds (TPCs) and the antioxidant activity of the carob pulp flour of the different varieties were determined by extracting these compounds in an ethanolic solution. Briefly, 1 g of carob pulp flour was added to 20 mL of ethanol 85% *v*/*v*. The resulting mixture was homogenized using an Ultra Turrax (Heidolph DIAX 600, Schwabach, Germany) for 1 min. The samples were stored in the refrigerator for 24 h and then centrifuged (ALC 4218 Thermo Fischer Scientific, Milan, Italy) at 2500 rpm for 5 min before being vacuum-filtered. The filtrate was stored in a centrifuge tube protected from light and kept in the refrigerator (4 °C) until analysis. The TPC was measured using the Folin–Ciocalteu assay, as described by Dalmau et al. [48], while the antioxidant activity was determined using the FRAP, ABTS, and CUPRAC assay, following the methodology of González-Centeno et al. [55,56]. Absorbance was recorded at 25 °C using a UV/Vis/NIR spectrophotometer (Thermo Scientific MultiSkan Spectrum, Vantaa, Finland) and correlated with standard calibration curves. TPC results were expressed as mg gallic acid equivalent per gram of carob pulp flour on a dry matter basis (dm), and the antioxidant activity results were expressed as mg Trolox equivalent per gram of CRF.

### 4.4. Statistical Analysis

Statistical analyses were performed using R software version 4.2.2 in conjunction with RStudio IDE [57]. The physicochemical and antioxidant properties of the cellulose-rich extracts, as well as the structural characteristics of the polysaccharides, were measured in triplicate. Each data point represents the average of three independent experiments.

Shapiro–Wilk test and the Levene test were used to assess the normality and homoscedasticity, respectively. When the data followed a normal distribution and the variances were homogeneous, a one-way ANOVA was applied to assess the existence of significant differences among the carob varieties. Tukey’s post hoc test was then used to determine the differences among the samples. Significant differences were considered when the *p*-value was smaller than 0.05.

## Figures and Tables

**Table 1:** Percentage (by weight) of pulp and seed in each of the different carob varieties.

Variety	% Pulp	% Seed
*Valenciana*	82.5	17.5
*Fulla de raó*	85.0	15.0
*Franco*	88.7	11.3
*Rotjal*	87.0	13.0
*Bugadera*	87.4	12.6
*De la mel*	84.4	15.6

**Table 2:** Moisture content (g H_2_O/100 g pulp), pH, acidity (g citric acid/100 g pulp), soluble sugars (g sucrose/100 g pulp), and cellulose-rich fraction (CRF, g/100 g pulp) yield of different carob varieties. Different letters indicate significant differences (*p* < 0.05) between carob varieties.

Variety	Moisture Content	pH	Acidity	Soluble Sugars	CRF
*Valenciana*	14.4 ± 0.1 (a)	5.23 ± 0.02 (a)	3.0 ± 0.4 (cd)	34.0 ± 1.5 (d)	45.6 ± 2.1 (ab)
*De la mel*	13.9 ± 0.8 (ab)	5.23 ± 0.01 (a)	4.4 ± 0.0 (a)	38.2 ± 0.3 (bc)	44.5 ± 2.0 (ab)
*Rotjal*	12.4 ± 0.1 (ab)	4.96 ± 0.04 (d)	2.3 ± 0.3 (d)	35.0 ± 1.8 (cd)	49.1 ± 1.9 (a)
*Bugadera*	13.2 ± 0.0 (ab)	4.88 ± 0.01 (e)	4.0 ± 0.2 (ab)	41.6 ± 0.6 (ab)	41.4 ± 2.1 (b)
*Franco*	11.6 ± 2.3 (ab)	5.16 ± 0.01 (b)	3.3 ± 0.4 (bc)	38.5 ± 0.2 (bc)	45.7 ± 2.3 (ab)
*Fulla de raó*	11.2 ± 0.8 (b)	5.10 ± 0.01 (c)	3.0 ± 0.4 (cd)	43.4 ± 2.7 (a)	40.9 ± 2.0 (b)

**Table 3:** Composition of the sugars in the cellulose-rich fraction (CRF) samples (values expressed as mg sugar/g CRE). Rha: rhamnose, Fuc: fucose, Ara: arabinose, Xyl: xylose, Man: mannose, Gal: galactose, Glc: glucose, and UA: uronic acids.

Variety	Rha	Fuc	Ara	Xyl	Man	Gal	Glc	UA
*Valenciana*	12.8 ± 0.1	9.1 ± 0.1	83.1 ± 4.6	134.7 ± 6.2	24.5 ± 3.9	55.9 ± 4.7	175.1 ± 13.6	146.1 ± 7.7
*De la mel*	9.1 ± 0.1	6.9 ± 0.1	70.8 ± 4.8	148.4 ± 7.5	22.7 ± 5.7	48.1 ± 8.0	168.8 ± 13.9	96.1 ± 6.5
*Rotjal*	11.4 ± 0.1	9.4 ± 0.1	84.7 ± 2.5	210.4 ± 5.7	31.6 ± 1.34	46.6 ± 0.3	209.6 ± 8.5	219.2 ± 10.3
*Bugadera*	11.1 ± 0.1	6.2 ± 0.1	74.7 ± 1.6	129.6 ± 10.4	20.3 ± 1.5	45.4 ± 0.7	149.6 ± 3.1	183.3 ± 7.3
*Franco*	11.3 ± 0.1	7.2 ± 0.1	78.9 ± 5.2	165.0 ± 11.3	25.1 ± 0.8	41.8 ± 1.1	189.6 ± 12.6	193.6 ± 11.3
*Fulla raó*	7.8 ± 0.1	6.6 ± 0.1	77.2 ± 2.2	161.3 ± 70.7	24.5 ± 4.3	46.4 ± 3.2	183.2 ± 10.9	95.5 ± 6.7

**Table 4:** Structural characteristics of the pectins in the CRF from different carob varieties. Different letters indicate significant differences (*p* < 0.05).

Variety	Linearity	Number	Length	DME (%)
*Valenciana*	1.0 ± 0.1 (b)	11.6 ± 2.0 (c)	11.2 ± 3.9 (a)	57.2 ± 1.7 (b)
*De la mel*	0.8 ± 0.0 (b)	10.6 ± 0.0 (c)	13.1 ± 2.2 (a)	56.6 ± 1.7 (b)
*Rotjal*	1.5 ± 0.1 (a)	19.3 ± 0.3 (ab)	11.6 ± 1.0 (a)	71.5 ± 2.1 (a)
*Bugadera*	1.4 ± 0.2 (a)	16.5 ± 2.2 (b)	10.9 ± 0.1 (a)	56.7 ± 1.7 (b)
*Franco*	1.5 ± 0.2 (a)	21.7 ± 1.3 (a)	11.1 ± 2.4 (a)	46.7 ± 1.4 (c)
*Fulla de raó*	0.7 ± 0.0 (b)	12.2 ± 0.3 (c)	15.8 ± 1.1 (a)	60.9 ± 1.8 (b)

**Table 5:** Total Phenolic Content (TPC mg gallic acid equivalent/g CRF) and antioxidant capacity measured by FRAP, ABTS, and CUPRAC methods (mg Trolox equivalent/g CRF) for different carob varieties. Different letters indicate significant differences (*p* < 0.05).

Variety	TPC	FRAP	ABTS	CUPRAC
*Valenciana*	38 ± 4 (b)	25 ± 3 (b)	610 ± 60 (b)	300 ± 30 (c)
*De la mel*	44 ± 4 (b)	49 ± 4 (a)	640 ± 80 (b)	670 ± 60 (a)
*Rotjal*	30 ± 3 (b)	21 ± 2 (b)	560 ± 60 (b)	250 ± 20 (c)
*Bugadera*	68 ± 6 (a)	56 ± 6 (a)	1070 ± 90 (a)	700 ± 70 (a)
*Franco*	37 ± 4 (b)	32 ± 3 (b)	480 ± 70 (b)	420 ± 50 (b)
*Fulla de raó*	38 ± 5 (b)	29 ± 4 (b)	590 ± 50 (b)	370 ± 40 (bc)

**Table 6:** General characteristics of the different varieties of carob analyzed.

Carob Variety	Photograph of the Fruit	Photograph of the Carob Tree	Shape	Type of Crop
*Bugadera*	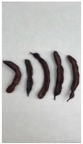	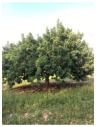	Elongated semi-curved	Irrigable land
*Fulla de raó*	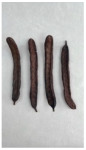	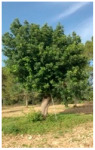	Elongated, thick	Dry farming
*Valenciana*	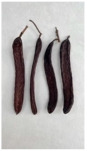	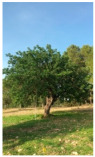	Elongated	Dry farming
*Rotjal*	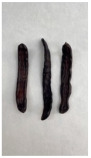	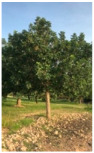	Elongated, thick	Dry farming
*Franco*	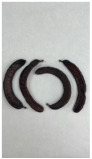	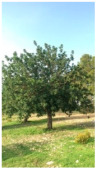	Elongated curved	Dry farming
*De la mel*	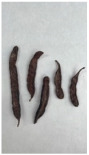	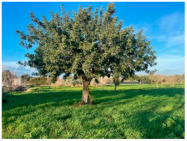	Thin, small-sized	Dry farming

## Data Availability

The original contributions presented in this study are included in the article. Further inquiries can be directed to the corresponding author.

## References

[1] Brassesco M.E., Brandão T.R.S., Silva C.L.M., Pintado M. (2021). Carob Bean (*Ceratonia siliqua* L.): A New Perspective for Functional Food. Trends Food Sci. Technol..

[2] Correia M.A., Romano P.J., Carob A., Benelli C., Ganino T., Giordano C., Petruccelli R., Beghé D., Martins-Loução M.A., Correia P.J. (2024). Carob: A Mediterranean Resource for the Future. Plants.

[3] Yousif A.K., Alghzawi H.M. (2000). Processing and Characterization of Carob Powder. Food Chem..

[4] El Batal H., Hasib A., Ouatmane A., Dehbi F., Jaouad A., Boulli A. (2016). Sugar Composition and Yield of Syrup Production from the Pulp of Moroccan Carob Pods (*Ceratonia siliqua* L.). Arab. J. Chem..

[5] Sȩczyk Ł., Świeca M., Gawlik-Dziki U. (2016). Effect of Carob (*Ceratonia siliqua* L.) Flour on the Antioxidant Potential, Nutritional Quality, and Sensory Characteristics of Fortified Durum Wheat Pasta. Food Chem..

[6] Frassoldati A., Ranzi E. (2019). Modeling of Thermochemical Conversion of Biomasses. Reference Module in Chemistry, Molecular Sciences and Chemical Engineering.

[7] Ebringerová A., Hromádková Z., Košťálová Z., Sasinková V. (2008). Chemical Valorization of Almond Shells.

[8] Willats W.G.T., Knox J.P., Mikkelsen J.D. (2006). Pectin: New Insights into an Old Polymer Are Starting to Gel. Trends Food Sci. Technol..

[9] Ström A., Ribelles P., Lundin L., Norton I., Morris E.R., Williams M.A.K. (2007). Influence of Pectin Fine Structure on the Mechanical Properties of Calcium-Pectin and Acid-Pectin Gels. Biomacromolecules.

[10] Lopez-Sanchez P., Martinez-Sanz M., Bonilla M.R., Wang D., Gilbert E.P., Stokes J.R., Gidley M.J. (2017). Cellulose-Pectin Composite Hydrogels: Intermolecular Interactions and Material Properties Depend on Order of Assembly. Carbohydr. Polym..

[11] Maruyama S., Streletskaya N.A., Lim J. (2021). Clean Label: Why This Ingredient but Not That One?. Food Qual. Prefer..

[12] Ioannou G.D., Savva I.K., Christou A., Stavrou I.J., Kapnissi-Christodoulou C.P. (2023). Phenolic Profile, Antioxidant Activity, and Chemometric Classification of Carob Pulp and Products. Molecules.

[13] Correa M.J., Salinas M.V., Carbas B., Ferrero C., Brites C., Puppo M.C. (2017). Technological Quality of Dough and Breads from Commercial Algarroba–Wheat Flour Blends. J. Food Sci. Technol..

[14] Loullis A., Pinakoulaki E. (2017). Carob as Cocoa Substitute: A Review on Composition, Health Benefits and Food Applications. Eur. Food Res. Technol..

[15] Aydın S., Özdemir Y. (2017). Development and Characterization of Carob Flour Based Functional Spread for Increasing Use as Nutritious Snack for Children. J. Food Qual..

[16] Vitali Čepo D., Mornar A., Nigović B., Kremer D., Radanović D., Vedrina Dragojević I. (2014). Optimization of Roasting Conditions as an Useful Approach for Increasing Antioxidant Activity of Carob Powder. LWT-Food Sci. Technol..

[17] Haddarah A., Ismail A., Bassal A., Hamieh T., Ioannou I., Ghoul M. (2013). Morphological and chemical variability of lebanese carob varieties. Eur. Sci. J..

[18] Llompart Salas B. (2023). Revalorització Dels Subproductes Derivats de La Comercialització de La Garrova (Ceratonia Síliqua L.): Fibra i Composts Antioxidants 2023.

[19] Adam I.A.A.F. (2019). Wild Fruits: Composition, Nutritional Value and Products.

[20] Tounsi L., Mkaouar S., Bredai S., Kechaou N. (2022). Valorization of Carob By-Product for Producing an Added Value Powder: Characterization and Incorporation into Halva Formulation. J. Food Meas. Charact..

[21] Benchikh Y., Paris C., Louaileche H., Charbonnel C., Ghoul M., Chebil L. (2017). Comparative Characterization of Green and Ripe Carob (*Ceratonia siliqua* L.): Physicochemical Attributes and Phenolic Profile. SDRP J. Food Sci. Technol..

[22] Sciammaro L.P. (2015). Caracterización Fisicoquímica de Vainas y Harinas de Algarrobo (*Prosopis Alba* y *Prosopis Nigra*). Ph.D. Thesis.

[23] Khlifa M., Bahloul A., Kitane S. (2013). Determination of Chemical Composition of Carob Pod (*Ceratonia siliqua* L) and Its Morphological Study. J. Mater. Environ. Sci..

[24] Nasar-Abbas S.M., E-Huma Z., Vu T.H., Khan M.K., Esbenshade H., Jayasena V. (2016). Carob Kibble: A Bioactive-Rich Food Ingredient. Compr. Rev. Food Sci. Food Saf..

[25] Femenia A., Sánchez E.S., Simal S., Rosselló C. (1999). Compositional Features of Polysaccharides from Aloe Vera (Aloe Barbadensis Miller) Plant Tissues. Carbohydr. Polym..

[26] Zhu B.J., Zayed M.Z., Zhu H.X., Zhao J., Li S.P. (2019). Functional Polysaccharides of Carob Fruit: A Review. Chin. Med..

[27] Papaefstathiou E., Agapiou A., Giannopoulos S., Kokkinofta R. (2018). Nutritional Characterization of Carobs and Traditional Carob Products. Food Sci. Nutr..

[28] Franz G., Blaschek W. (1990). 8—Cellulose. Methods Plant Biochem..

[29] Kumar Gupta P., Sai Raghunath S., Venkatesh Prasanna D., Venkat P., Shree V., Chithananthan C., Choudhary S., Surender K., Geetha K. (2019). An Update on Overview of Cellulose, Its Structure and Applications. Cellulose.

[30] Szymanska-Chargot M., Chylinska M., Gdula K., Koziol A., Zdunek A. (2017). Isolation and Characterization of Cellulose from Different Fruit and Vegetable Pomaces. Polymer.

[31] Umaña M.M., Dalmau M.E., Eim V.S., Femenia A., Rosselló C. (2019). Effects of Acoustic Power and PH on Pectin-Enriched Extracts Obtained from Citrus by-Products. Modelling of the Extraction Process. J. Sci. Food Agric..

[32] Gunning A.P., Bongaerts R.J.M., Morris V.J. (2009). Recognition of Galactan Components of Pectin by Galectin-3. FASEB J..

[33] Khamsucharit P., Laohaphatanalert K., Gavinlertvatana P., Sriroth K., Sangseethong K. (2017). Characterization of Pectin Extracted from Banana Peels of Different Varieties. Food Sci. Biotechnol..

[34] Prakash Maran J., Sivakumar V., Thirugnanasambandham K., Sridhar R. (2013). Optimization of Microwave Assisted Extraction of Pectin from Orange Peel. Carbohydr. Polym..

[35] Velásquez-Arredondo H.I., Ruiz-Colorado A.A., De Oliveira S. (2010). Ethanol Production Process from Banana Fruit and Its Lignocellulosic Residues: Energy Analysis. Energy.

[36] Rodríguez-González V.M., Femenia A., González-Laredo R.F., Rocha-Guzmán N.E., Gallegos-Infante J.A., Candelas-Cadillo M.G., Ramírez-Baca P., Simal S., Rosselló C. (2011). Effects of Pasteurization on Bioactive Polysaccharide Acemannan and Cell Wall Polymers from Aloe Barbadensis Miller. Carbohydr. Polym..

[37] Petkova N., Petrova I., Ivanov I., Mihov R., Hadjikinova R., Ognyanov M., Nikolova V. (2017). Nutritional and Antioxidant Potential of Carob (*Ceratonia siliqua*) Flour and Evaluation of Functional Properties of Its Polysaccharide Fraction. J. Pharm. Sci. Res..

[38] Ganji F., Vasheghani Farahani S., Vasheghani-Farahani E. (2010). Theoretical Description of Hydrogel Swelling: A Review. Iran. Polym. J..

[39] Blažic R., Marušić K., Vidović E. (2023). Swelling and Viscoelastic Properties of Cellulose-Based Hydrogels Prepared by Free Radical Polymerization of Dimethylaminoethyl Methacrylate in Cellulose Solution. Gels.

[40] Elleuch M., Bedigian D., Roiseux O., Besbes S., Blecker C., Attia H. (2011). Dietary Fibre and Fibre-Rich by-Products of Food Processing: Characterisation, Technological Functionality and Commercial Applications: A Review. Food Chem..

[41] Tofanica B.M., Mikhailidi A., Samuil C., Ungureanu O.C., Fortună M.E., Ungureanu E. (2024). Advances in Cellulose-Based Hydrogels: Current Trends and Challenges. Gels.

[42] Ortega N., Macià A., Romero M.-P., Trullols E., Morello J.-R., Anglès N., Motilva M.-J., Ortega N., Macià A., Romero M.-P. (2009). Rapid Determination of Phenolic Compounds and Alkaloids of Carob Flour by Improved Liquid Chromatography Tandem Mass Spectrometry. J. Agric. Food Chem..

[43] Saci F., Bachir Bey M., Louaileche H., Gali L., Bensouici C. (2020). Changes in Anticholinesterase, Antioxidant Activities and Related Bioactive Compounds of Carob Pulp (*Ceratonia siliqua* L.) during Ripening Stages. J. Food Meas. Charact..

[44] Rtibi K., Selmi S., Grami D., Saidani K., Sebai H., Amri M., Eto B., Marzouki L. (2017). *Ceratonia siliqua* L. (Immature Carob Bean) Inhibits Intestinal Glucose Absorption, Improves Glucose Tolerance and Protects against Alloxan-Induced Diabetes in Rat. J. Sci. Food Agric..

[45] International Standard International Organization for Standardization Meat and Meat Products-Determination of Total Fat Content. https://cdn.standards.iteh.ai/samples/6038/6b692ef064484574aec2816f3343ed68/ISO-1443-1973.pdf.

[46] AOAC Método Oficial AOAC 932.14 Sólidos En Almíbar. http://files.foodmate.com/2013/files_2967.html.

[47] Dalmau M.E., Bornhorst G.M., Eim V., Rosselló C., Simal S. (2017). Effects of Freezing, Freeze Drying and Convective Drying on in Vitro Gastric Digestion of Apples. Food Chem..

[48] Coimbra M.A., Delgadillo I., Waldron K.W., Selvendran R.R. (1996). Isolation and Analysis of Cell Wall Polymers from Olive Pulp. Mod. Methods Plant Anal..

[49] González-Centeno M.R. (2013). Caracterización de Los Subproductos de la Industria Vitivinícola Como Fuente De Fibra Dietética Y Compuestos Fenólicos. Uso de Los Ul-Trasonidos De Potencia Para La Extracción de la Fracción Fenólica.

[50] Wang W., Ma X., Xu Y., Cao Y., Jiang Z., Ding T., Ye X., Liu D. (2015). Ultrasound-Assisted Heating Extraction of Pectin from Grapefruit Peel: Optimization and Comparison with the Conventional Method. Food Chem..

[51] Kaya M., Sousa A.G., Crépeau M.-J., Sørensen S.O., Ralet M.-C. (2014). Characterization of Citrus Pectin Samples Extracted under Different Conditions: Influence of Acid Type and PH of Extraction. Ann. Bot..

[52] Manrique G.D., Lajolo F.M. (2002). FT-IR Spectroscopy as a Tool for Measuring Degree of Methyl Esterification in Pectins Isolated from Ripening Papaya Fruit. Postharvest Biol. Technol..

[53] Kuniak L., Marchessault R.H. (1972). Study of the Crosslinking Reaction between Epichlorohydrin and Starch. Starch.

[54] González-Centeno M.R., Rosselló C., Simal S., Garau M.C., López F., Femenia A. (2010). Physico-Chemical Properties of Cell Wall Materials Obtained from Ten Grape Varieties and Their Byproducts: Grape Pomaces and Stems. LWT-Food Sci. Technol..

[55] González-Centeno M.R., Comas-Serra F., Femenia A., Rosselló C., Simal S. (2015). Effect of Power Ultrasound Application on Aqueous Extraction of Phenolic Compounds and Antioxidant Capacity from Grape Pomace (*Vitis vinifera* L.): Experimental Kinetics and Modeling. Ultrason. Sonochem..

[56] González-Centeno M.R., Knoerzer K., Sabarez H., Simal S., Rosselló C., Femenia A. (2014). Effect of Acoustic Frequency and Power Density on the Aqueous Ultrasonic-Assisted Extraction of Grape Pomace (*Vitis vinifera* L.)–A Response Surface Approach. Ultrason. Sonochem..

[57] RStudio Team (2022). RStudio: Integrated Development for R.

